# Validation of ERICVA Risk Score as a Predictor of One Year Amputation-Free Survival of Patients with Critical Limb Ischemia

**DOI:** 10.1016/j.avsg.2021.02.013

**Published:** 2021-08

**Authors:** Sara-Azhari Mohamed, Navian Lee Viknaswaran, Jonathan Doran, Clara Sanz-Nogués, Khalid Ahmed, Linda Howard, Muhammad Tubassam, Timothy O'Brien, Stewart Redmond Walsh

**Affiliations:** 1 Regenerative Medicine Institute, National University of Ireland Galway, Ireland; 2 Department of Vascular Surgery, Galway University Hospital, Galway, Ireland; 3 School of Medicine, National University of Ireland Galway, Ireland; 4 College of Medicine, University of Limerick, Ireland; 5 Department of Vascular Surgery, Southmead Hospital NHS Foundation Trust, Bristol, UK; 6 School of Medicine, University College London, London, UK; 7 Department of Endocrinology, Galway University Hospital, Ireland; 8 Department of Vascular Surgery, National University of Ireland Galway, Ireland

**Keywords:** Critical Limb Ischemia, ERICVA score, Risk Score

## Abstract

**Background:**

The ERICVA score was derived to predict amputation-free survival in patients with critical limb ischemia (CLI). It may be a useful tool to stratify patients in trials of novel interventions to treat CLI but, as yet, it has not been externally validated.

**Methods:**

A prospective database of CLI patients was developed during prescreening of patients for a phase 1 stem cell therapy clinical trial. The primary outcome was amputation free survival (AFS) at 1 year. Both the full ERICVA scale (11 parameters) and simplified ERICVA scale (5 parameters) were validated. Data analysis was performed by calculation of the area under the receiver operating characteristic (ROC) curve examining the predictive value of the scores. The Chi-square test was used to examine the association between risk group and one-year AFS and the cumulative survival of the three risk groups was compared using Kaplan Meier survival curves.

**Results:**

A series of 179 CLI patients were included in the analysis. The Chi-square test of independence showed a significant association between the risk group (high, medium and low) and one-year AFS outcome (*P = 0.*0007). Kaplan-Meier survival curve showed significant difference in one-year AFS between the three risk groups (log-rank *P < 0.*001). The area under the curve (AUC) was found to be 0.63 and 0.61 for the full and simplified score, respectively. The sensitivity of the full score was 0.44 with specificity of 0.84. The simplified score had a sensitivity of 0.28 and specificity of 0.92.

**Conclusion:**

The ERICVA risk score system was found to have a fair validity but cannot be considered reliable as a single predictor of one year AFS of CLI patients. The simplified score had an AUC almost identical to the full score and can accordingly replace the full score.

## Introduction

Critical Limb Ischemia (CLI) is the most advanced manifestation of peripheral vascular disease (PAD), with annual incidence of 0.4%.^1^ It represents a disease of high morbidity and mortality. Revascularization is the preferred therapy, usually achieved through surgical or endovascular interventions. However, the short and medium term risk of amputation and/or death after revascularization remains high.^2^ Therefore, objective assessment of risk benefit/ratio is required prior to intervention.

Several risk scoring systems have been proposed to help surgeons in selecting patients who would benefit from revascularization from those who require primary amputation or palliative care with best medical therapy.3., 4., 5., 6.. Nevertheless, most of these scores lack external validation in different populations and therefore have limited clinical applicability.^7^

The ERICVA score (*Escala de Riesgo en Isquemia Critica de Valladolid,* Valladolid Critical Limb Ischemia Risk Scale) was derived in a tertiary hospital in Spain to predict amputation-free survival (AFS) in patients with CLI.^7^ The score was derived from a sample of 561 CLI cases and validated internally on a sample of 111 cases, where it was reported to be better than the Finnvasc & PREVENT III scores.^8^^,^^9^ It may be a useful tool for prediction of prognosis and possibly stratification of patients in trials of novel interventions to treat CLI, but as yet, it has not been externally validated. The purpose of this study is to validate the ERICVA scale in predicting AFS in patients with CLI.

## Methods

A prospective database of CLI patients was developed in a tertiary hospital at the West of Ireland during prescreening of patients for a phase 1 stem cell therapy clinical trial.^10^ Both the full (Table I) and simplified (Table II) ERICVA scales were validated. The cohort included patients diagnosed with CLI, regardless of their eligibility for revascularization. CLI was defined as persistent rest pain and/or tissue loss (Rutherford Class 4, 5 or 6) in the form of ulceration or gangrene. In each patient, age, sex, Rutherford classification and medical comorbidities were registered from hospital medical records (paper and/or digital). The different parameters of both full and simplified scores were collected and according to the overall score in each scale, patients were classified as low, medium or high risk (Tables I and II).

**Table I:** ERICVA scale.

ERICVA risk scale	Value
Cerebrovascular disease	5 points
Previous contralateral major amputation	5 points
Diabetes mellitus	3 points
Dialysis	9 points
COPD/asthma	6 points
Active cancer in the previous 5 years	12 points
Hematocrit <30%	9 points
Neutrophil/lymphocyte ratio ≥5	8 points
Absent perimalleolar Doppler signal	6 points
Urgent admission	3 points
Rutherford class 6 (major tissue loss)	9 points

Low risk (0-9 points), Mild risk (10-19 points), High risk (>19 points).

**Table II:** Simplified ERICVA scale*.

Simplified ERICVA risk scale	Value
Dialysis	1 point
Active Cancer in the previous 5 years	1 point
Hematocrit > 30%	1 point
Neutrophil/lymphocyte ratio ≥ 5	1 point
Rutherford class 6	1 point

Low risk (0 points), Mild risk (1 point), High risk (>1 point).

⁎ Created from the five parameters with the greatest weight in the ERICVA scale.

The primary outcome was defined as amputation free survival (AFS) at 1 year. Descriptive statistical analysis was performed using Chi-Square test of Independence to determine association between risk group and one-year AFS. Kaplan-Meier survival curves were used to determine if the difference in cumulative one-year amputation-free survival between the three risk groups was significant. To evaluate the validity of the score, the area under the receiver operating characteristic (ROC) curve was calculated for both the full and the simplified score. All statistical analyses were performed using StatsDirect 3 (Llumina Press 2010).

The study protocol was approved by the Research Ethics Committee at Galway University Hospital and General Data Protections Regulations were followed.

## Results

**Fig. 1:**
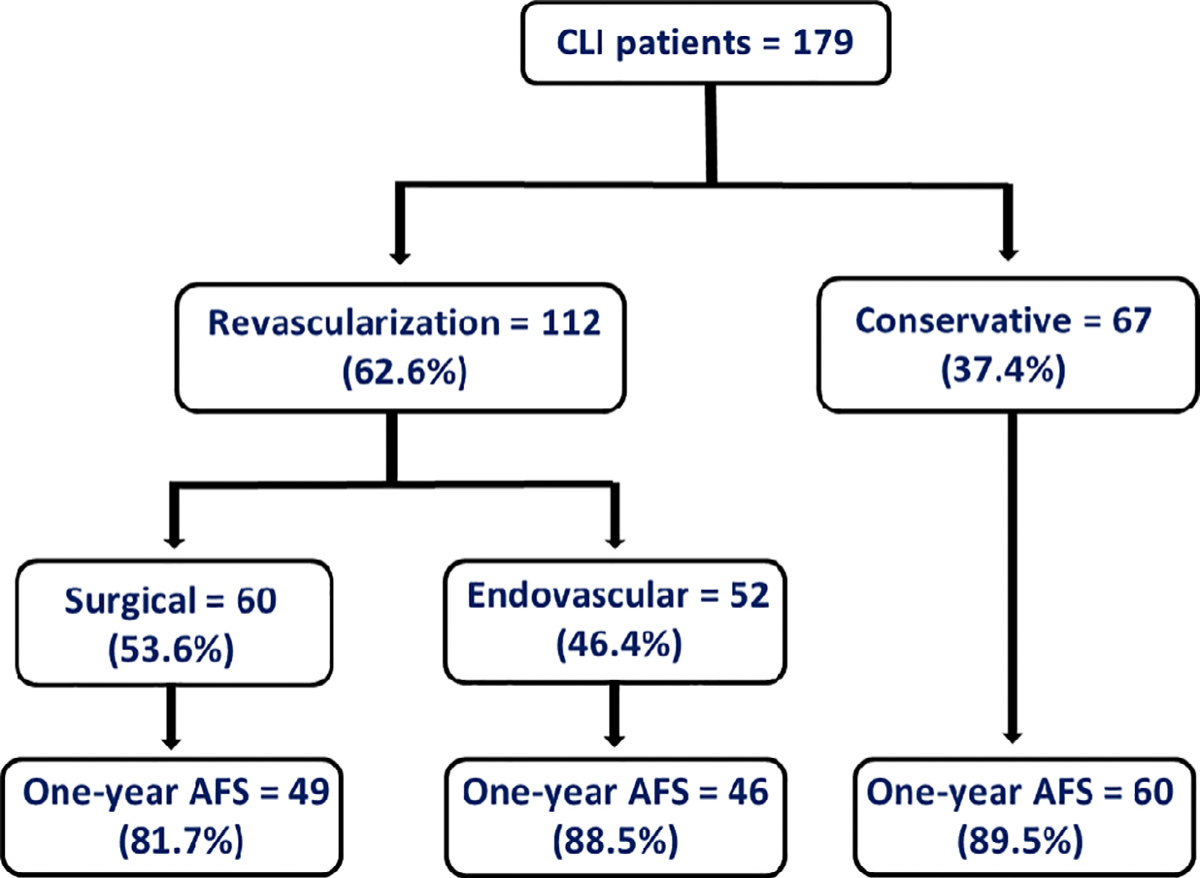
Study Flow Chart showing the proportion of patients treated conservatively versus those treated with endovascular or surgical interventions and the one-year amputation-free survival for each group.

A total of 179 CLI patients were included in the study, 62.6% (112) of whom underwent revascularization and 37.4% (67) were treated conservatively (Fig. 1). The majority of patients were males (72.1%), with a median age of 70 years (range: 28 to 93). The demographics and comorbidities of this population are compared with those of the population used to internally validate the score system^7^ in Table III. This comparison revealed significant difference in patients’ mean age, Rutherford class 6, rate of urgent admission, vessel calcification, diabetes, smoking status, and history of contralateral revascularization or major amputation (Table III).

**Table III:** Comparison of patient's clinical characteristics from two studies.

	Brizuela-Sanz et al. *N* = 111	Mohamed et al. N = 179	*P*-value
Sex (males), *n* (%)	91 (82)	129 (72.1)	0.066
Age (years), mean (SD)	73.5 (10.4)	70.1 (11.8)	0.011*
Urgent admission, *n* (%)	76 (68.5)	101 (56.4)	0.048*
Rutherford class, *n* (%)			
6	40 (36)	12 (6.7)	0.000*
5	53 (47.7)	122 (68.1)	0.001*
4	18 (16.2)	45 (25.1)	0.080
Vessel calcification (ABI<1.3), *n* (%)	38 (34.2)	19 (10.6)	0.000*
Hypertension, *n* (%)	77 (69.4)	114 (63.7)	0.373
Diabetes mellitus, *n* (%)	62 (55.9)	68 (38)	0.004*
Smoker, *n* (%)	72 (64.9)	85 (47.5)	0.005*
Cerebrovascular disease, *n* (%)	14 (12.6)	26 (14.5)	0.728
Ischemic heart disease, *n* (%)	41 (36.9)	63 (35.2)	0.802
COPD/asthma, *n* (%)	18 (16.2)	18 (10.1)	0.143
Dialysis, *n* (%)	4 (3.6)	7 (3.9)	1.000
Cancer, *n* (%)	13 (11.7)	20 (11.2)	1.000
Previous contralateral major amputation	13 (11.7)	5 (2.8)	0.004*
Previous contralateral revascularization	28 (25.2)	113 (63.1)	0.000*
Previous major revascularization (same limb)	37 (33.3)	70 (39.1)	0.381
Preoperative blood tests, mean (SD)			
Hematocrit (%)	37.93 (5.88)	38.7 (5.8)	0.277
Neutrophils (× 10 ^9^/L)	6.21 (2.79)	6.8 (3.6)	0.119
Lymphocytes (× 10 ^9^/L)	1.77 (0.81)	1.80 (1.0)	0.780
Neutrophil/lymphocyte ratio	4.5 (3.87)	4.92 (4.4)	0.395

ABI, ankle brachial index; COPD, chronic obstructive pulmonary disease; N, sample size, *n*, frequency; SD= standard deviation; * *P*-value < 0.05.

**Fig. 2:**
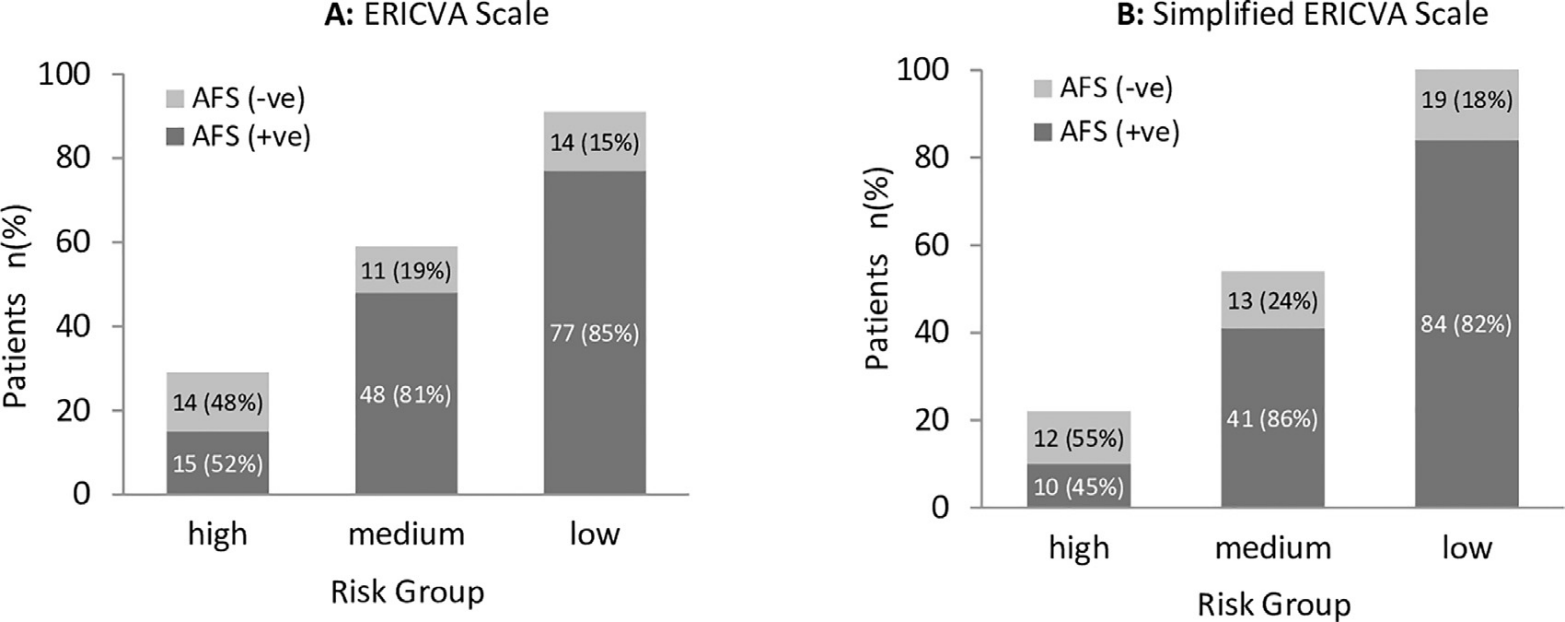
One-year amputation-free survival (AFS) for each risk group. **A.** ERICVA scale: low (0–9 points), medium (10–19 points), and high (20 or more points), Total χ^2^ = 14.47, (*P 0*.0007). **B.** Simplified ERICVA scale: low (0 points), medium (1 point), and high (2 or more points), Total χ^2^ = 11.74, (*P*0.0028).

For both the full and the simplified ERICVA scores, results showed that higher scores were associated with lower rates of one-year AFS (Fig. 2). The Chi-square test of independence showed a significant association between the risk group and one-year AFS outcome (χ^2^ = 14.47, *P* .0007 and χ^2^ = 11.74, *P* 0.0028 for the full and simplified score respectively).

**Fig. 3:**
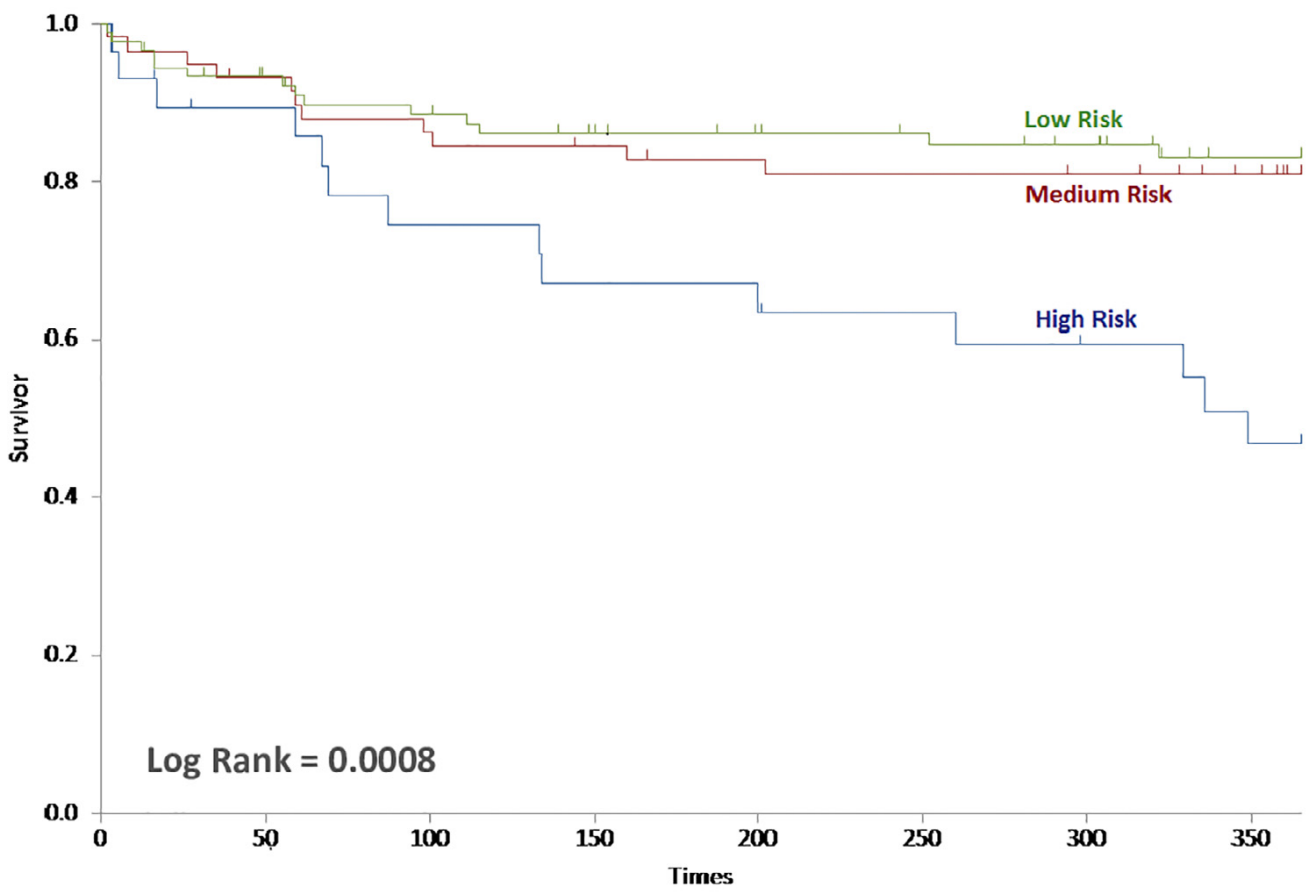
Kaplan-Meier curve of one-year amputation-free survival of the Full ERICVA score stratified by risk level (log-rank = 0.0008).

**Fig. 4:**
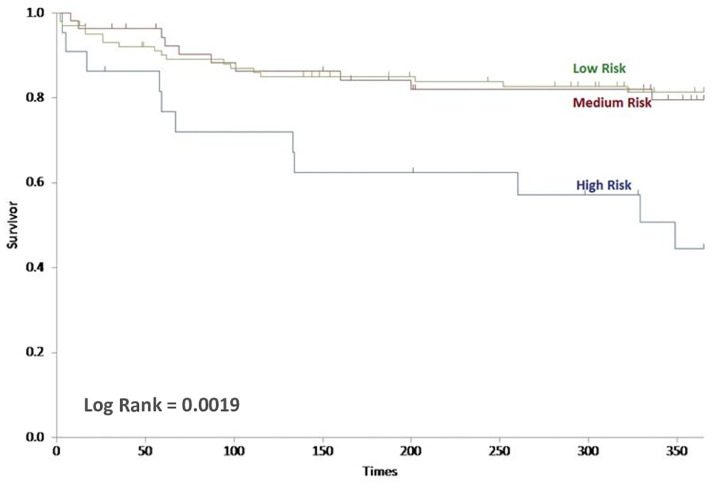
Kaplan-Meier curve of one-year amputation-free survival of the Simplified ERICVA score stratified by risk level (log-rank = 0.0019).

For the full ERICVA score, Kaplan Meier survival analysis demonstrated that the primary outcome of mean one-year AFS was 319, 313 and 253 days in the low, medium and high risk groups (Fig. 3). The mean overall AFS was 690, 680 and 455 days for the low, medium and high risk groups; respectively (*P* = 0.0008; log rank test). For the simplified score, Kaplan Meier survival analysis demonstrated that the primary outcome of mean one-year AFS was 314, 314 and 243 days in the low, medium and high-risk groups (Fig. 4). The mean overall AFS was 676, 667 and 443 days for the low, medium and high risk groups; respectively (*P* = 0.0019; log rank test).

**Fig. 5:**
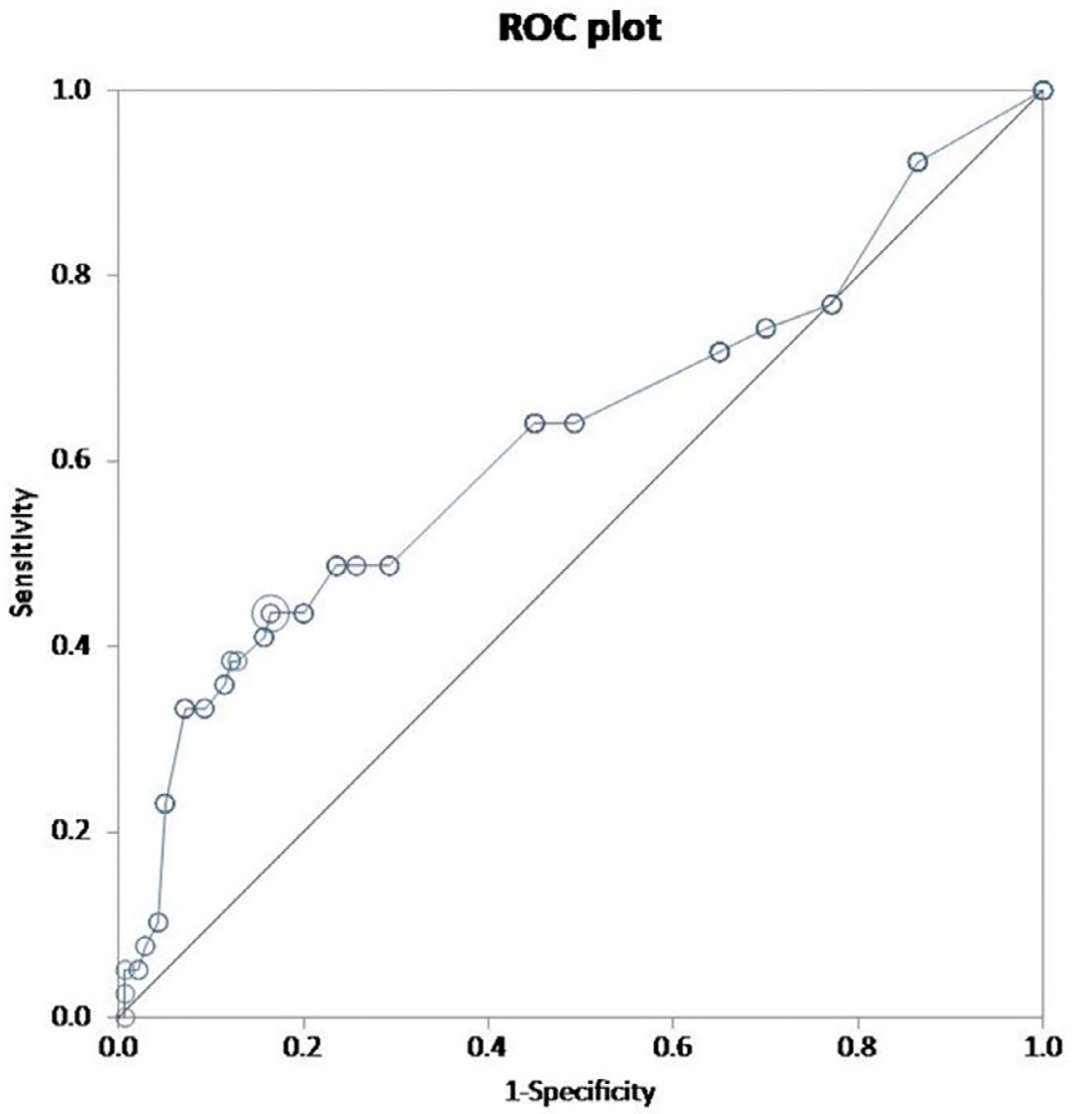
Receiver operator curve of the full ERICVA score. Validity of the ERICVA score evaluated by calculation of the area under the receiver operating curve (ROC) using the extended trapezoidal rule. Selected optimum cut-off point = 16. Area under ROC curve by = 0.626 (95% CI = 0.516 to 0.737). sensitivity = 0.436 (95% CI = 0.278 to 0.604). specificity = 0.836 (95% CI = 0.764 to 0.893).

**Fig. 6:**
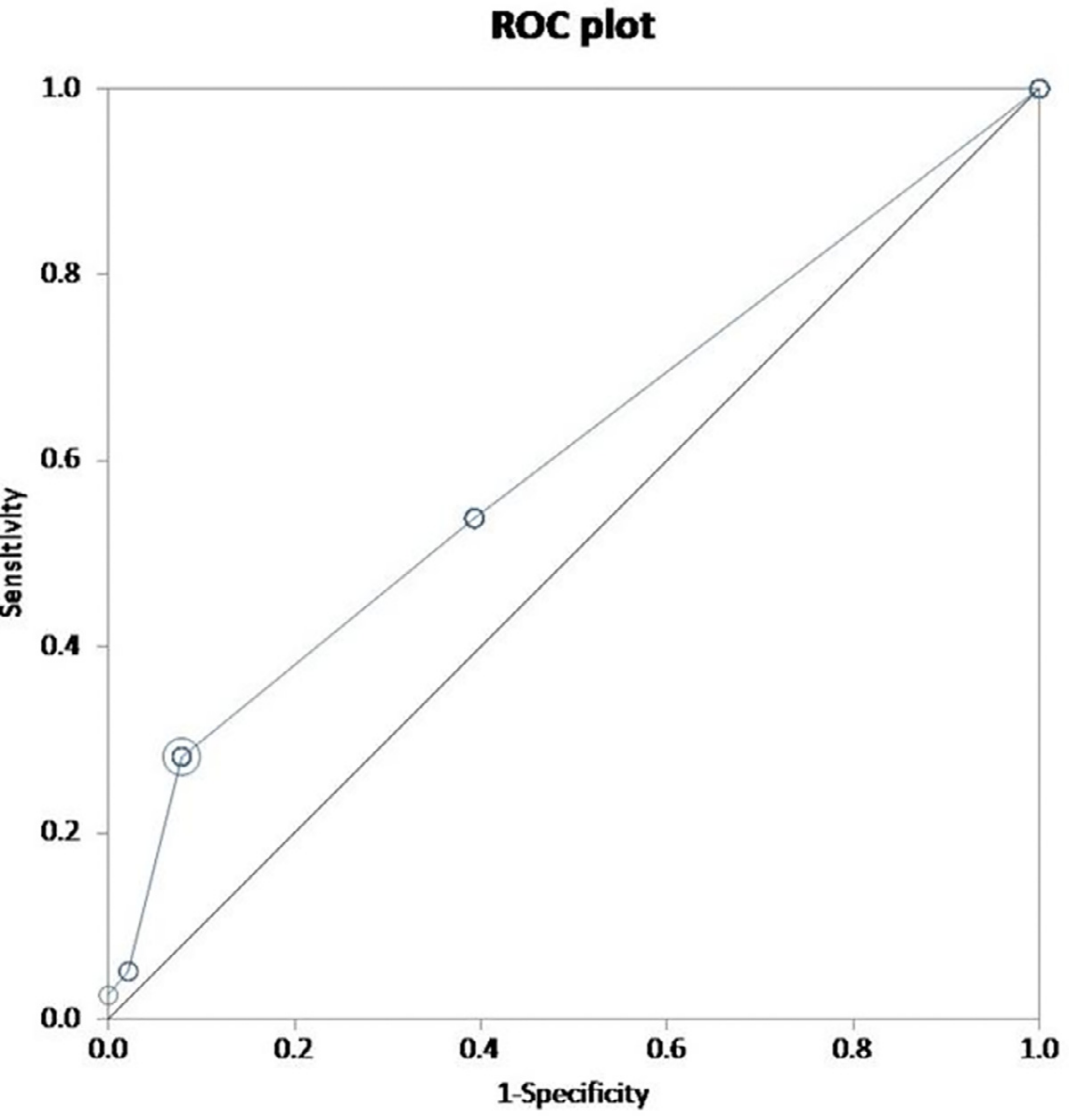
Validity of the ERICVA score evaluated by calculation of the area under the receiver operating curve (ROC) using the extended trapezoidal rule. Selected optimum cut-off point = 2. Area under ROC curve by = 0.606 (95% CI = 0.508 to 0.705). sensitivity = 0.282 (95% CI = 0.150 to 0.449). specificity = 0.921 (95% CI = 0.864 to 0.960).

To determine the sensitivity and specificity of the scores, ROC curves were constructed. The AUC was found to be 0.63 and 0.61 for the full and simplified score, respectively (Figs. 5 and 6). For an optimum cut-off point of 16, the sensitivity of the full score was 0.44 (95% CI: 0.28 to 0.60) and specificity 0.84 (95% CI: 0.76 to 0.89). The simplified score had a sensitivity of 0.28 (95% CI: 0.15 to 0.45) and specificity 0.92 (95% CI: 0.86 to 0.96); with 2 as the optimum cut-off point. Table IV provides the sensitivity and specificity for different cut-off points for both the full and the simplified ERICVA scores.

**Table IV:** Sensitivity and specificity of the different cut-off point for the receiver operator curve.

	Cut-off point	Sensitivity	Specificity
Full ERICVA score	10	0.64	0.55
	15	0.43	0.80
	16*	0.44	0.84
	17	0.39	0.87
	21	0.33	0.91
	24	0.23	0.95
Simplified ERICVA score	1	0.54	0.61
	2*	0.28	0.92
	3	0.03	1.00

⁎ Youden index (*J*) optimum cut-off point.

## Discussion

Surgical or endovascular revascularization is currently considered the gold standard in treatment of patients with critical limb ischemia. Despite the great improvement in technology and interventional devices, revascularization is still associated with significant risk of morbidity and mortality. Objective risk/ benefit assessment of CLI patients is required to select patients who are suitable for intervention. Moreover, several clinical trials are testing stem cell therapy as a novel treatment for CLI.11., 12., 13., 14.. Patient stratification is essential to ensure proper selection of patients for those clinical trials.

The current study focused on externally validating the ERICVA risk scoring system, as it is the most recently proposed scoring system for CLI, with performance shown to be superior to both the Finnvasc and PREVENT III scores.^7^ The score was developed from a cohort of more than 500 patients and internally validated in a cohort of 111 cases. Appropriate methodology was used to develop the score by selecting variables associated with death and/or major amputation for the multivariate Cox regression analysis. A total of 11 variables were used to develop the full score and the 5 factors with the highest weight for the simplified score.

While the ERICVA score was derived and internally validated on revascularized CLI patients; in this study, the score was validated on patients who were treated either conservatively or via revascularization. This approach was followed as the role of the scoring system is essential for decision making prior to determination of the optimal treatment option. The results of this study also demonstrate comparable rates of one-year AFS for patients treated conservatively and those treated with surgical or endovascular intervention (Fig. 1).

The results of this study on CLI patients in Ireland supports the external validity of the ERICVA score system as a predictor of one year AFS. However, the calculated AUC for the studied cohort was lower than the reported by the score developers. This difference may be related to the observed significant difference in the baseline characteristics between the two populations; with the developer's cohort showing more advanced disease (Rutherford class 6 and contralateral major amputation) and higher prevalence of smoking and diabetes.

The scores had high specificity but very low sensitivity. As the validity of the full score was found to be identical to the simplified score, the use of the latter provides a practical easy-to-apply scoring system for CLI. However, the score should be used with caution as the sensitivity and specificity do not support its use as a single predictor of one-year AFS. The results also support the use of the score system to predict long-term AFS in CLI patients, with the cumulative survival inversely proportional to the risk score.

## Conclusion

The ERICVA risk score system has fair validity but cannot be considered reliable as a single predictor of one year AFS of CLI patients. The simplified score had an AUC almost identical to the full score and can accordingly replace the full score, providing a reliable alternative risk assessment system.

## Funding

• Science Foundation Ireland /Health Research Board (SFI/HRB) Translational Research Award, Award number: TRA 201115.
• NUIG, School of Medicine PhD Scholarship.
• HRB Clinical Research Facility Galway CRFG.
• Wellcome Trust Vacation Scholarship 2016 (Ref No: 202224/Z/16/Z).

## Authors Names and Their Contribution

**Sara Azhari Mohamed**: Conception and design, administrative support, collection and assembly of data, data analysis and interpretation, manuscript writing.

**Navian Lee Viknaswaran:** Collection and assembly of data, data analysis.

**Jonathan Doran:** Collection and assembly of data, manuscript writing.

**Clara Sanz-Nogués:** Data analysis and interpretation, manuscript writing.

**Khalid Ahmed:** Data analysis and interpretation.

**Linda Howard**: Manuscript writing, final approval of the manuscript.

**Muhammad Tubassam**: Conception and design, provision of study material or patients, collection and assembly of data.

**Timothy O'Brien**: Conception and design, administrative support, final approval of the manuscript.

**Stewart Redmond Walsh**: Conception and design, administrative support, Provision of study material or patients, collection and assembly of data, data analysis and interpretation, manuscript writing, final approval of the manuscript.
